# Comparative transcriptome analysis of genes and metabolic pathways involved in sporulation in *Ganoderma lingzhi*

**DOI:** 10.1093/g3journal/jkab448

**Published:** 2022-01-17

**Authors:** Manjun Cai, Zengdong Tan, Xiaoxian Wu, Xiaowei Liang, Yuanchao Liu, Yizhen Xie, Xiangmin Li, Chun Xiao, Xiong Gao, Shaodan Chen, Huiping Hu, Qingping Wu

**Affiliations:** Guangdong Provincial Key Laboratory of Microbial Safety and Health, State Key Laboratory of Applied Microbiology Southern China, Institute of Microbiology, Guangdong Academy of Sciences, Guangzhou 510070, China; National Key Laboratory of Crop Genetic Improvement, Huazhong Agricultural University, Wuhan 430070, China; Guangdong Provincial Key Laboratory of Microbial Safety and Health, State Key Laboratory of Applied Microbiology Southern China, Institute of Microbiology, Guangdong Academy of Sciences, Guangzhou 510070, China; Guangdong Provincial Key Laboratory of Microbial Safety and Health, State Key Laboratory of Applied Microbiology Southern China, Institute of Microbiology, Guangdong Academy of Sciences, Guangzhou 510070, China; Guangdong Provincial Key Laboratory of Microbial Safety and Health, State Key Laboratory of Applied Microbiology Southern China, Institute of Microbiology, Guangdong Academy of Sciences, Guangzhou 510070, China; Guangdong Yuewei Edible Fungi Technology Co. Ltd., Guangzhou 510663, China; Guangdong Provincial Key Laboratory of Microbial Safety and Health, State Key Laboratory of Applied Microbiology Southern China, Institute of Microbiology, Guangdong Academy of Sciences, Guangzhou 510070, China; Guangdong Yuewei Edible Fungi Technology Co. Ltd., Guangzhou 510663, China; Guangdong Provincial Key Laboratory of Microbial Safety and Health, State Key Laboratory of Applied Microbiology Southern China, Institute of Microbiology, Guangdong Academy of Sciences, Guangzhou 510070, China; Guangdong Provincial Key Laboratory of Microbial Safety and Health, State Key Laboratory of Applied Microbiology Southern China, Institute of Microbiology, Guangdong Academy of Sciences, Guangzhou 510070, China; Guangdong Provincial Key Laboratory of Microbial Safety and Health, State Key Laboratory of Applied Microbiology Southern China, Institute of Microbiology, Guangdong Academy of Sciences, Guangzhou 510070, China; Guangdong Provincial Key Laboratory of Microbial Safety and Health, State Key Laboratory of Applied Microbiology Southern China, Institute of Microbiology, Guangdong Academy of Sciences, Guangzhou 510070, China; Guangdong Provincial Key Laboratory of Microbial Safety and Health, State Key Laboratory of Applied Microbiology Southern China, Institute of Microbiology, Guangdong Academy of Sciences, Guangzhou 510070, China; Guangdong Provincial Key Laboratory of Microbial Safety and Health, State Key Laboratory of Applied Microbiology Southern China, Institute of Microbiology, Guangdong Academy of Sciences, Guangzhou 510070, China

**Keywords:** *Ganoderma lingzhi*, sporulation, meiosis, transcriptional regulation, carbohydrate metabolism, coexpression network

## Abstract

Over the past decades, *Ganoderma lingzhi* spores have received considerable attention as a great potential pharmaceutical resource. However, the genetic regulation of sporulation is not well understood. In this study, a comparative transcriptome analysis of the low-sporing HZ203 and high-sporing YW-1 was performed to characterize the mechanism underlying sporulation. A total of 917 differentially expressed genes were identified in HZ203 and 1,450 differentially expressed genes in YW-1. Differentially expressed genes involved in sporulation were identified, which included *HOP1*, *Mek1*, *MSH4*, *MSH5*, and *Spo5* in meiosis. Positive regulatory pathways of sporulation were proposed as 2 transcriptional factors had high connectivity with *MSH4* and *Spo5*. Furthermore, we found that the pathways associated with energy production were enriched in the high-sporing genotype, such as the glyoxylate and dicarboxylate metabolism, starch and sucrose metabolism. Finally, we performed a weighted gene coexpression network analysis and found that the hub genes of the module which exhibit strong positive relationship with the high-sporing phase purportedly participate in signal transduction, carbohydrate transport and metabolism. The dissection of differentially expressed genes during sporulation extends our knowledge about the genetic and molecular networks mediating spore morphogenesis and sheds light on the importance of energy source during sporulation.

## Introduction

“Lingzhi” is a medicinal mushroom that has been renowned for more than 2,000 years in China and has been assigned to *Ganoderma lucidum* for over a century. Based on morphological and molecular studies, a new species *Ganoderma lingzhi* has been proposed for “Lingzhi” (Cao et al. 2012; Dai et al. 2017). A breakthrough in the artificial cultivation of *G. lingzhi* has been achieved since the 1950s (Li et al. 2019b), which lays the critical foundation for scientific research on the identity and function of the medicinal metabolites. *G. lingzhi* spores, mature germ cells generated from diploid nuclei, which undergo 2 rounds of meiosis (Coelho et al. 2017), showed a variety of beneficial pharmacological effects (Li et al. 2017; Na et al. 2017; Jiao et al. 2020a; Liu et al. 2021).

Despite progress in understanding the biological effects and underlying mechanism of action of *G. lingzhi* spores (GLS), the genetic regulation of sporulation remains largely unknown. In cultivated mushrooms, sporulation-deficient (sporeless) mutants have been investigated to identify causal genes of the sporeless phenotype (Okuda et al. 2009, 2012, 2013; Lavrijssen et al. 2020); and the well-known genes are the homologs of *MSH4*. *MSH4* plays a key role in recombination in human (Snowden et al. 2004); *MSH4* homologs *stpp1* (Okuda et al. 2013) and *poMSH4* (Lavrijssen et al. 2020) are responsible for sporulation in *Pleurotus pulmonarius* and *Pleurotus ostreatus*, respectively. In micromycetes, reports on genes involved in sporulation indicate that genes in the meiotic process are necessary for sporulation, for example, the involvement of *rad9* in chromosome condensation (Seitz et al. 1996), *MRE11* in homolog pairing (Gerecke and Zolan 2000), *SPO11* and *stpp1* in recombination (Baudat and de Massy 2004; Okuda et al. 2013), and *Mek1* in double-strand break repair (Hollingsworth and Gaglione 2019). In our previous study, developmental transcriptome analyses of 3 sporulation stages in *G. lingzhi* were performed using RNA sequencing (RNA-seq) and we found that several genes encoding apparent homologs of the factors involved meiotic process were upregulated during sporulation (Cai et al. 2021). Since spore morphogenesis is generated from diploid nuclei that undergo 2 rounds of meiosis (Coelho et al. 2017), genes that participate in meiosis might be involved in the control of sporulation in *G. lingzhi*. However, our knowledge of sporulation is still limited.

RNA-seq is used to unravel complex biological processes and has been successfully utilized in fungi (Krizsán et al. 2019; Yoo et al. 2019; Huang et al. 2020). Weighted correlation network analysis (WGCNA) is a system biology method for describing the correlation patterns among genes across microarray samples and can be used to find clusters (modules) of highly correlated genes. Furthermore, WGCNA can be employed to construct gene networks where each node represents a gene, and the connecting lines (edges) between genes represent coexpression correlations. Genes that showed that the most interconnections in the network were assigned as hub genes (Langfelder and Horvath 2007, 2008). WGCNA has also been used to identify hub genes at different developmental stages in Chinese cordyceps (*Ophiocordyceps sinensis*, syn. *Cordyceps sinensis*) (Li et al. 2019a), and the key pathways and genes involved in blight fruiting body formation in *Flammulina velutipes* (Wang et al. 2019). Recently, the lignocellulose, carbohydrate, and triterpenoid contents of *G. lingzhi* were integrated with enzyme expression levels using WGCNA, which indicated that the synthesis of triterpenoids can be enhanced by regulating the expression of enzymes in the triterpenoid pathway, in carbohydrate metabolism and substrate degradation (Zhou et al. 2021). These studies indicate that WGCNA can be used to explore the underlying networks and candidate genes of certain traits in fungi.

In the present study, comparative transcriptome analysis of 2 contrasting *G. lingzhi* genotypes (high sporing in YW-1 and low sporing in HZ203) was performed. We identified several structural genes and transcription factors (TFs), which might function in meiosis; moreover, hub genes positively associated with sporulation were found to function in energy production, carbohydrate transport and metabolism. Our data help emphasize the role of meiosis and carbohydrate metabolism on basidiosporogenesis in *G. lingzhi* and provide a valuable source for elucidating the genetic regulatory networks of sporulation.

## Materials and methods

### *G. lingzhi* strains and sampling

*G. lingzhi* dikaryotic strains YW-1 and HZ203 from the Institute of Microbiology, Guangdong Academy of Sciences were selected for our study. HZ203 is a low-sporing strain, while YW-1 produces numerous spores during fruiting body development. After 20 days of growth on sorghum medium at 25°C, mycelia were inoculated onto the substrate packed in heat-sealed cultivation bags with microfilter windows and cultured in the dark at 25°C for 1 month. For fruiting body growth, the bags were cultured in a room with 10 h of illumination and 30 min of ventilation at 26°C. GLS development was divided into 3 stages according to their fruiting body morphology and spore number. Fruiting bodies in the first stage are the youngest, with a large area of white edge, without pores or spores. Those in the second stage begin to show obvious pores in the center of the abaxial side of the pileus and have several spores near every pore. Mature fruiting bodies with numerous spores in YW-1 and a few spores in HZ203 were assigned to the third stage. The transcriptional dynamics of genes during these 3 sporulation stages (YW1, YW2, and YW3) in YW-1 have been reported in our previous study (Cai et al. 2021). Given that the spore number at the second stage did not differ significantly between YW-1 and HZ203, samples at the first (YW1, HZ1) and third stages (YW3, HZ2) were selected for comparative analysis. Abaxial sides of the pileus from at least 3 independent bags with the same developmental stage were selected and quickly frozen in liquid nitrogen. Three replicate samples were prepared for each developmental stage. All samples were stored at −80°C prior to RNA isolation.

### Cytological observation

The pileus from transverse and longitudinal sections of YW-1 and HZ203 at the third developmental stage (YW3 and HZ2) were fixed in 2.5% glutaraldehyde overnight at 4°C, washed using phosphate-buffered saline 3 times for 10 min each, dehydrated in a graded series of ethanol for 15 min each (30%, 50%, 70%, 85%, 95%, and 100% ethanol), with ethanol replaced with 100% tertiary butanol twice for 20 min each; the sections were freeze-dried, observed, and photographed using a Hitachi S-3000N scanning electron microscope (Hitachi, Chiyoda-Ku, Tokyo, Japan).

### Total RNA extraction and sequencing

Total RNA was extracted from different pileus samples using the TRIzol Kit (Invitrogen, Carlsbad, CA, USA), according to the manufacturer’s instructions. RNA degradation and contamination were monitored using 1% agarose gels. RNA concentration was assessed using a Nanodrop 2.0 (Thermo Fisher Scientific, Waltham, MA, USA) and Agilent 2100 Bioanalyzer (Agilent, Santa Clara, CA, USA). Sequencing libraries were generated according to the Illumina kit VAHTS mRNA-seq V3 Library Prep Kit for Illumina (Vazyme Biotech, Nanjing, China). Finally, Illumina HiSeq mRNA sequencing was used for high-throughput sequencing with paired-end 150-bp reads (BioMarker Technology, Beijing, China).

### Quantification of gene expression level

A total of 15 RNA-seq data sets were used in the study, of which 9 from 3 developmental stages of YW-1 were deposited into the Sequence Read Archive database (accession number: PRJNA704770) and have been published in a previous study (Cai et al. 2021) and 6 from 2 developmental stages of HZ203 were generated in this study and also deposited (accession number: PRJNA736534). Raw RNA-seq reads were trimmed with Trimmomatic v.0.36 (Bolger et al. 2014) (parameters: ILLUMINACLIP:TruSeq3-PE. fa:2:30:10:LEADING:3 TRAILING:3 SLIDINGWINDOW:4:20 MINLEN:50). Next, clean reads were mapped to the reference genome (Chen et al. 2012) using HISAT2 (Kim et al. 2015) (parameters: –dta -p 6 –max-intronlen 5000000) and then String Tie (Pertea et al. 2015) was used to calculate the gene expression level. Fragments per kilobase of transcript per million fragments mapped (FPKM) of each gene was calculated based on the length of the gene and reads count mapped to this gene (Pertea et al. 2015); the FPKM values represented the expression level of each transcript.

### Differential expression analysis and gene annotation

Differential expression analysis between mature fruiting bodies with spores and young fruiting bodies without spores (YW3/YW1, HZ2/HZ1) was performed using the DESeq2 package (Love et al. 2014) to identify differentially expressed genes (DEGs), and the adjusted *P*-values were calculated using the Benjamini–Hochberg method to control the false discovery rate (FDR). FDR <0.01, and fold change ≥2 were set to screen out the DEGs between each set of compared samples. DEG sequences were blasted using Gene Ontology (GO), Clusters of Orthologous Groups of proteins (COG), Kyoto Encyclopaedia of Genes and Genomes (KEGG), Eukaryotic Orthologous Groups of proteins (KOG), Pfam, Swiss-Prot, eggNOG (evolutionary genealogy of genes: Non-Supervised Orthologous Groups), and NR (Non-Redundant Protein Sequence Database) databases using BLAST software.

### Coexpression network analysis

Gene coexpression networks were constructed using the WGCNA package in the R software (Langfelder and Horvath 2008). The parameter value determination for module construction was 13 for this dataset, and a total of 16 modules were formed according to the pairwise correlations of gene expression across all samples and coexpression patterns of individual genes. Network visualization for “brown” module was performed using the Cytoscape software version 3.5.1, with a cutoff of the weight parameter obtained from the WGCNA set at 0.4 (Shannon et al. 2003).

### Validation of DEG expression by qRT-PCR

Total RNA extracted from all samples subjected to transcriptome analysis was used for qRT-PCR. Single-stranded cDNA was synthesized from 1 μg of total RNA using HiScript II Q RT SuperMix for qRT-PCR (+gDNA wiper) (Vazyme Biotech, Nanjing, China), following the manufacturer’s instructions. Gene-specific primers were designed using Primer3 Input and are listed in Supplementary Table 1. qRT-PCR was performed using the Applied Biosystems ABI 7500 (Applied Biosystems, Foster City, CA, USA), and the 2 × AceQ qPCR SYBR Green Master Mix (Vazyme Biotech, Nanjing, China) was added to the reaction system according to the manufacturer’s instructions. All genes were run in triplicate from 3 biological replicates. The internal reference gene, *18 S*, was used to normalize the expression data. Relative expression was determined using the 2^−ΔΔCT^ method (Livak and Schmittgen 2001).

## Results

### Spore phenotypic characterization of 2 *G. lingzhi* strains, YW-1 and HZ203

**Fig. 1.**
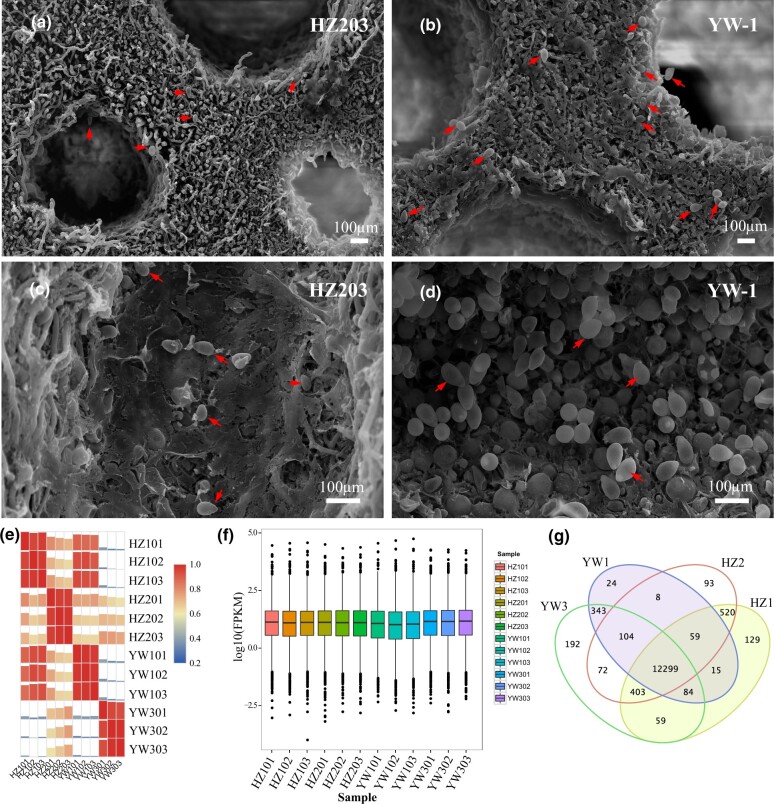
Spores on the transverse and longitudinal sections and the summary of transcriptome sequence datasets. Comparisons of the spore number on the transverse a, b) and longitudinal c, d) sections of mature fruiting body in the YW-1 and HZ203. Red arrows indicate the spores. Scale bar = 100 μm. e) Pearson correlation analysis based on global RNA-seq data from 12 libraries. f) Comparison of gene expression levels among different experimental groups. g) Venn diagram shows the number of sample-specific expressed genes and the shared expression genes between samples.

YW-1 and HZ203 are 2 cultivated *G. lingzhi* strains in China. At the mature stage, spores ejected from the pileus of YW-1 were well above the number of HZ203. To examine the developmental difference between these 2 strains, we observed the abaxial side of the pileus by scanning electron microscopy and found that the pores of both strains developed well (Supplementary Fig. 1), thus excluding defective pore development as an underlying cause. Simultaneously, we observed that the spores on the transverse and longitudinal sections in HZ203 (Fig. 1, a and c) were scarce compared to YW-1 (Fig. 1, b and d), which indicated that limited spore morphogenesis contributed to the less spore ejection at the mature stage in HZ203.

### Differences between sporulation stages were more evident than that between strains at transcriptional level

To explore potential molecular mechanisms underlying differences on spore morphogenesis during sporulation of the 2 *G. lingzhi* strains, RNA-seq analysis was conducted on the abaxial side of the pileus to generate transcriptional profiles. Samples from fruiting bodies without spores (HZ1 and YW1), and nearly mature fruiting bodies with numerous spores in YW-1 (YW3) and a few spores in HZ203 (HZ2) were collected. After removing low-quality reads and adaptor sequences, approximately 86.28% of reads could be mapped to the reference genome (Supplementary Table 2). In addition, 375 and 393 genes identified in YW-1 and HZ203, respectively, were not found in the monokaryotic strain G.260125-1 (Supplementary Table 3). The expression pattern among biologically repeated samples was highly consistent, and the correlation coefficient values between stages was larger than that between strains (Fig. 1e), indicating that differences between 2 developmental stages were evident at the transcriptional level and good repeatability among biological replicates.

Gene expression levels among different experimental groups were compared (Fig. 1f) and results showed that most genes (80.6%) were moderately expressed, with FPKM values ranging from 1 to 100 (Supplementary Table 4). The highly (FPKM values >100) and lowly (FPKM values <1) expressed genes accounted for 9.6% and 9.8%, respectively (Supplementary Table 4). Genes with FPKM values <1 were excluded from the subsequent analysis. In total, 12,299 genes were expressed in all experimental groups, and the specific genes expressed in YW1, YW3, HZ1, and HZ2 were 24, 192, 129, and 93, respectively (Fig. 1g). Approximately 88.5% of the detected genes, 13,662 in YW-1 and 13,845 in HZ203, could be functionally annotated by aligning the gene sequences to public protein databases (Table 1).

**Table 1.** Functional annotation of *G. lingzhi* deduced proteins by sequence similarity search.

Annotated_Database	Strains	Annotated_Number		300 ≤ length < 1,000		Length ≥ 1,000	
		All	New-isoform	All	New-isoform	All	New-isoform
OG_Annotation	YW-1	3,831	17	928	9	2,886	8
	HZ203	3,850	18	939	9	2,894	9
GO_Annotation	YW-1	5,502	72	1,639	33	3,779	39
	HZ203	5,510	67	1,636	29	3,784	37
KEGG_Annotation	YW-1	3,709	44	1,136	21	2,512	23
	HZ203	3,709	39	1,126	18	2,518	21
KOG_Annotation	YW-1	4,985	29	1,268	12	3,685	17
	HZ203	5,008	28	1,277	12	3,697	16
Pfam_Annotation	YW-1	7,207	49	1,992	17	5,164	32
	HZ203	7,231	50	2,003	19	5,177	31
Swiss-Prot_Annotation	YW-1	5,721	37	1,472	16	4,202	21
	HZ203	5,744	35	1,475	16	4,220	19
eggNOG_Annotation	YW-1	9,183	123	2,820	39	6,254	84
	HZ203	9,238	126	2,856	42	6,268	84
NR_Annotation	YW-1	12,093	373	4,318	146	7,513	225
	HZ203	12,239	391	4,396	150	7,549	234
All_Annotated	YW-1	12,109	375	4,327	147	7,518	226
	HZ203	12,255	393	4,405	151	7,553	235

### Differential response of transcriptional factors involved in spore morphogenesis

**Fig. 2.**
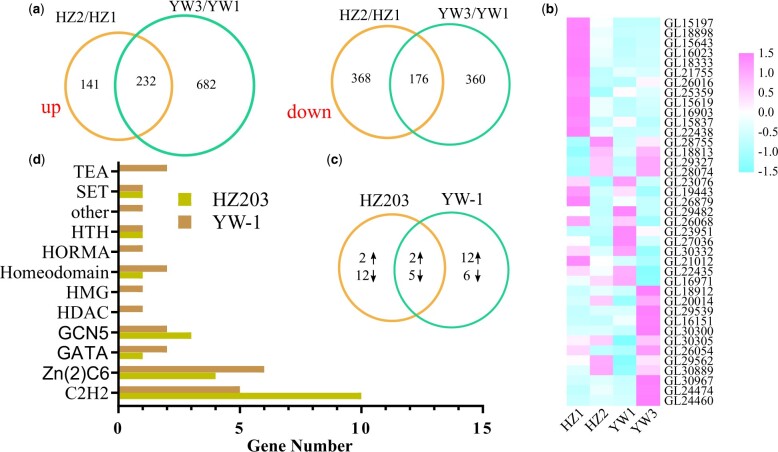
Expression of genes coding for transcription factors. a) Venn diagrams of differentially expressed transcripts between different developmental stages and the number of common DEGs in 2 strains. The left Venn diagram indicates the upregulated genes and the right one represents the downregulated genes. b) Heatmap of developmentally regulated TF-coding genes. c) Venn diagram of developmentally regulated TF-coding genes in YW-1 and HZ203. d) Transcription factor family distribution of developmentally regulated genes in YW-1 and HZ203.

To investigate the transcriptome differences between strains during sporulation, DEGs were analyzed. Compared to the young fruiting bodies without spores, 917 and 1,450 genes were differentially expressed at the mature fruiting bodies with spores in HZ203 and YW-1, respectively (Supplementary Table 5). In particular, the upregulated gene number in YW-1 was 4.8-fold higher than that in HZ203, while the number of downregulated genes was not significantly different (Fig. 2a). GO, COG, KEGG, KOG, Pfam, Swiss-Prot, eggNOG, and NR databases were used to annotate the functions of the DEGs (Supplementary Table 5). Approximately 90% of the DEGs were annotated using the databases (Table 2). To confirm the results from computational analysis, 10 DEGs were used for qRT-PCR analysis to assess their expression patterns in 2 strains during sporulation. The expression trends of the selected genes were consistent with the RNA-seq data (Supplementary Fig. 2), indicating the reliability of our transcriptome data.

**Table 2.** Number of differentially expressed genes annotated in multiple public databases.

Annotated_Database	HZ2/HZ1	YW3/YW1
COG	271	429
GO	300	465
KEGG	119	205
KOG	220	369
NR	817	1,300
Pfam	436	656
Swiss-Prot	318	507
eggnog	571	885
Total	821	1,302

TFs integrate environmental and developmental cues to fine-tune target gene expression, thus modulating cellular functions (John et al. 2021; Li et al. 2021). In this study, 39 TFs were differentially expressed in at least 1 comparative analysis (Supplementary Table 6 and Fig. 2b). Interestingly, 81% of the differentially expressed TFs in HZ203 were downregulated, a contrast to the expression profiles of TFs in YW-1 (Fig. 2c). Conserved domain annotation showed that 23 of 39 TFs belonged to the zinc finger family (Supplementary Table 6 and Fig. 2d), including the C2H2/Zn(2)Cys6/GATA subfamilies, which have been shown to play an essential role in sporulation (Carrillo et al. 2017). From the annotated results from the Pfam and Swiss-Prot databases, 5 genes, including *GL24474*, *GL16971*, *GL21755*, *GL26016*, and *GL23076* (Supplementary Table 6), were homologous with transcriptional regulators which are considered to be involved in spore morphogenesis. For example, GL24474 was homologous to the meiosis-specific protein, HOP1 (Hollingsworth et al. 1990), and its expression level was positively correlated with the extent of spore morphogenesis. These results indicated that GL24474 might play a role in the meiosis and thus involving in the control of sporulation in *G. lingzhi*.

In addition to the well-known cell cycle-related TFs, 2 Zn2Cys6 transcriptional activators of carbohydrate metabolism were identified, including the homologs of ethanol-regulated transcription factor 1 (ERT1) and xylanolytic transcriptional activator (XlnR). ERT1 is a transcriptional activator that regulates gluconeogenesis (Gasmi et al. 2014). The homolog of *ERT1* in *G. lingzhi* was *GL16151*, which was upregulated only in YW-1 during sporulation (Fig. 2b). XlnR is a transcriptional activator of xylanolytic and cellulolytic genes in *Aspergillus* (Marui et al. 2002; Noguchi et al. 2009). *GL30300*, the homolog of *XlnR*, was upregulated nearly 10-fold in YW-1 but was transcribed stably in HZ203 (Fig. 2b). The elevated expression levels of *GL16151* and *GL30300* might activate their target genes, thereby promoting the production of energy and structural components which are required for high sporing.

### Functional classification of differentially expressed genes in YW-1 and HZ203

**Fig. 3.**
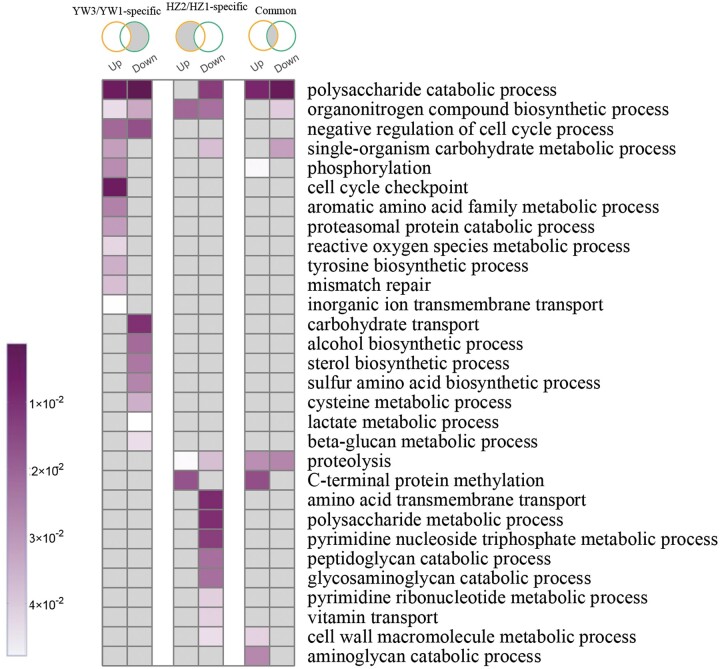
GO functional enrichment of differentially expressed genes. Venn diagrams represent DEGs among different comparisons (from left to right): DEGs specific in YW3/YW1; DEGs specific in HZ2/HZ1; and DEGs shared in HZ2/HZ1 and YW3/YW1. The GO terms with *P*-value <0.05 are shown in color; otherwise, they are in gray.

To characterize the biological roles of DEGs during sporulation, GO enrichment analysis was performed. Considering that spore morphogenesis occurs in both strains, common DEGs between strains might be involved in the control of spore morphogenesis, while the strain-specific DEGs might be caused by differences in the number of spores of the genotypes and their genetic make-up. As shown in Fig. 3, strain-specific DEGs were relevant to different classification of biological processes. For example, upregulated DEGs in YW-1 tend to be relevant to “cell cycle checkpoint,” “mismatch repair,” “proteasomal protein catabolic process,” and “phosphorylation,” while downregulated DEGs in HZ203 were enriched in “amino acid transmembrane transport” and “polysaccharide metabolic process.”

**Fig. 4.**
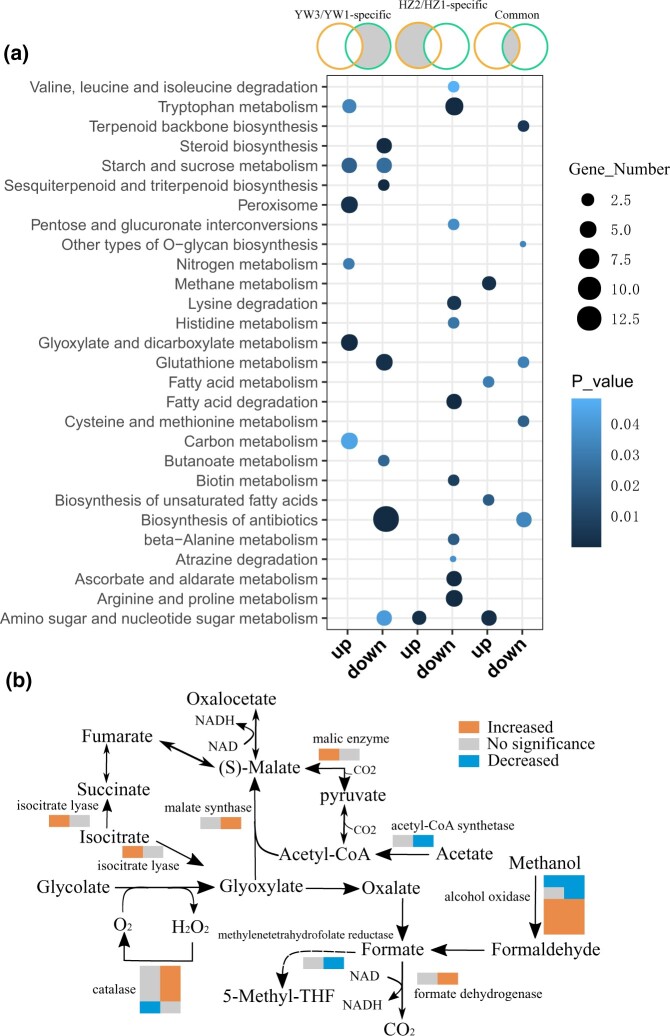
KEGG pathway enrichment of differentially expressed genes. a) KEGG enrichment for DEGs between different comparisons (from left to right): DEGs specific in YW3/YW1; DEGs specific in HZ2/HZ1; DEGs shared in HZ2/HZ1 and YW3/YW1. Significantly enriched pathways with *P* < 0.05 are shown. b) Differentially expressed genes in the carbon metabolic pathway. Two color bars for each enzyme from left to right represent the expression changes in HZ203 and YW-1, respectively.

Furthermore, DEGs in different subgroups were subjected to KEGG pathway enrichment analysis to elucidate the key metabolic pathways they might be involved in. As shown in Fig. 4a, a shift in pathway enrichment was observed in strain-specific and common DEGs. Carbohydrate metabolism pathways were upregulated in YW-1 as compared to HZ203, including the “glyoxylate and dicarboxylate metabolism,” “starch and sucrose metabolism,” and “carbon metabolism” (Fig. 4a). Analyzing DEGs in each pathway, we found that most genes involved in “methane,” “tryptophan,” “peroxisome,” and “glyoxylate and dicarboxylate metabolism” also played a role in “carbon metabolism” (Supplementary Table 7). To further elucidate the differences between strains, we identified all DEGs in carbon metabolism, as shown in Fig. 4b. Notably, genes in the glyoxylate and dicarboxylate metabolism pathway, which produces NADH, were upregulated in YW-1, including 3 catalase, 1 malate synthase, and 1 formate dehydrogenase-encoding gene (Fig. 4b). However, only isocitrate lyase and malic enzyme-encoding genes were upregulated in HZ203 cells. These results suggested that NADH derived from carbon metabolism, especially from glyoxylate and dicarboxylate metabolism pathway, might be one of the important energy sources for spore morphogenesis, thus increasing the spore yield in YW-1. The HZ203 specific downregulated genes were enriched in amino acid metabolism and degradation, including “arginine and proline metabolism,” “β-alanine metabolism,” “histidine metabolism,” “tryptophan metabolism,” “valine, leucine and isoleucine degradation,” and “lysine degradation” (Fig. 4a). Amino acids are suggested to be positive correlated with spore formation (Wang et al. 2013), which might explain fewer spores were formed at the mature stage in HZ203.

Contrary to the upregulated carbohydrate metabolism pathway, secondary metabolism, such as “sesquiterpenoid and triterpenoid biosynthesis,” “terpenoid backbone biosynthesis,” and “steroid metabolism” was downregulated (Fig. 4a). Expression patterns of genes involved in these pathways are shown in Supplementary Fig. 3a. In HZ203, the expression of genes encoding the rate-limiting enzyme 3-hydroxy-3-methylglutaryl CoA reductase (HMGR) and farnesyl-diphosphate synthase (FPS-2) was significantly decreased (Supplementary Fig. 3b). In addition to HMGR and FPS-2, the expression of genes encoding 3-hydroxy-3-methylglutaryl CoA synthase (HMGS), squalene synthase (SQS), squalene monooxygenase, and sterol 24-*C*-methyltransferase (ERG6) was significantly downregulated in YW-1 cells (Supplementary Fig. 3b). As the expression level of genes encoding enzymes was higher in HZ203 (Supplementary Fig. 3a), more acetyl-CoA, the precursor for the synthesis of triterpenoids, might be required, which would reduce the key metabolic intermediate into tricarboxylic acid cycle.

### Coexpression network analysis identified sporulation-related DEGs

**Fig. 5.**
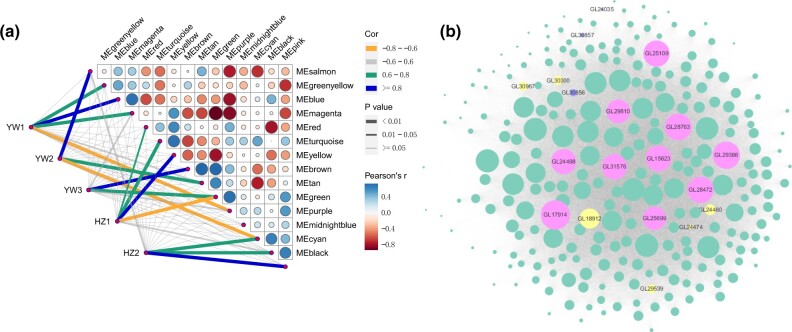
Coexpression network of transcripts involved in sporulation in *G. lingzhi*. a) Analysis of the module–trait association. The line color corresponds to the correlation coefficient between the module and the stage, the line weight corresponds to the *P*-value, circle size and color indicate the Pearson correlation between modules and is displayed according to the scale on the right panel. b) Correlation network analysis of the “brown” module. The gene coexpression network is a scale-free weighted gene network with multiple nodes connected to different nodes via edges. Each node represents a gene, and the size of node circle is positively correlated with the number of interacting genes. The hub genes are denoted in pink, the TF-coding genes are denoted in yellow, the meiosis-related genes are denoted in purple, and the other DEGs in this module are denoted in green.

To identify potential genes (structural genes and putative transcriptional factors) highly associated with spore morphogenesis, WGCNA analysis was performed. This analysis obtained 16 distinct modules as shown in the dendrogram (Supplementary Fig. 4), and the number of DEGs in each module ranged from 1 in the “midnightblue” to 467 in the “brown” (Supplementary Table 8). Furthermore, correlations between the modules and distinct samples were calculated, and all modules, except “midnightblue,” were significantly (*P <* 0.01) associated with at least 1 sample (Fig. 5a). Notably, the “brown” module had the highest positive correlation with YW3 (Fig. 5a), indicating that genes in this module might play an essential role in promoting spore morphogenesis and development.

According to the gene function annotation, 6 genes in the “brown” module were homologous to meiosis-related genes, which are considered to play a role in sporulation. For example, *GL24474* encodes a HORMA domain-containing protein that is highly similar to the meiosis-specific protein HOP1 in *Saccharomyces cerevisiae* (Hollingsworth et al. 1990; Kariyazono et al. 2019). In budding yeast, the regulation of meiotic double-strand break repair is controlled by a meiosis-specific kinase called Mek1 (Hollingsworth and Gaglione 2019), and the homology of Mek1 in *G. lingzhi* was GL30283. In addition, 3 genes, *GL30856*, *GL30857*, and *GL16852*, encode proteins that show high sequence similarity to the MutS homolog (MSH) family. MSH4 was the homolog of GL30856 and GL30857, whereas the homolog of GL16852 was MSH5. These 5 genes were upregulated in YW-1 but showed a constant expression pattern in HZ203 (Supplementary Table 8). Finally, the homolog of GL24035 is sporulation-specific protein 5 (Spo5) in *Schizosaccharomyces pombe*. Spo5, a putative meiosis-specific RNA-binding protein, is essential for meiotic progression, and the disruption of *spo5* causes abnormal sporulation, generating inviable spores due to failed forespore membrane formation and the absence of a spore wall (Kasama et al. 2006). Notably, *GL24035* was upregulated in both YW-1 and HZ203, and its expression level at the YW3 stage was more than twice as high at the HZ2 stage, which corresponded to the spore yield in YW-1 and HZ203.

TFs are well known to play a crucial role in sporulation; thus, we searched for the TFs in the “brown” module and 7 genes encoding C2H2-containing TFs (GL18912 and GL29539), Zn(2)C6-containing TF (GL30300 and GL16151), TEA domain-containing TF (GL24460), HORMA domain-containing TF (GL24474), and Homeobox domain-containing TF (GL30967) were identified (Supplementary Table 8). Each of these TFs interacted with several target genes and might regulate their expression (Fig. 5b). Notably, GL18912 (C2H2-containing TF) had high connectivity with *MSH4* (*GL30856* and *GL30857*) and *Spo5* (*GL24035*). In addition, GL24460 (TEA domain-containing TF) was highly connected with GL30856 (Supplementary Table 9).

Furthermore, 10 hub genes linked to this module were analyzed (Fig. 5b), and they encode different proteins, including the general substrate transporter (MFS, GL25109), glycosyl transferase (GL24498), Ser-Thr-rich glycosyl-phosphatidyl-inositol-anchored membrane protein (GL31576), serine/threonine-protein kinase (GL29810), NADP-dependent alcohol dehydrogenase (GL25699), and 5 hypothetical proteins (GL17914, GL29386, GL28763, GL28472, GL15623) (Table 3). KEGG enrichment analysis showed that the most significantly enriched pathways in “brown” module were consistent with the enrichment results in the YW-1 specific upregulated genes, suggesting that carbohydrate metabolism contributed to the enhanced spore yield (Supplementary Fig. 5).

**Table 3.** The hub genes detected in the “brown” and “pink” module.

Module	Gene_ID	Pfam/Swiss_Prot_annotation	eggNOG_class_annotation
Brown	GL17914	NA	Function unknown
	GL29386	NA	Function unknown
	GL28763	NA	Function unknown
	GL25109	Major Facilitator Superfamily	Carbohydrate transport and metabolism
	GL28472	NA	Function unknown
	GL15623	NA	Function unknown
	GL24498	Glycosyl transferase family 8	Posttranslational modification, protein turnover, chaperones
	GL31576	Ser-Thr-rich glycosyl-phosphatidyl-inositol-anchored membrane family	Function unknown
	GL29810	Serine/threonine-protein kinase	Signal transduction mechanisms
	GL25699	Alcohol dehydrogenase GroES-like domain; Zinc-binding dehydrogenase	Energy production and conversion
Pink	GL21769	NA	NA
	GL17422	NA	NA
	GL15200	Caspase domain	Posttranslational modification, protein turnover, chaperones
	GL26510	NA	NA
	GL24251	NA	RNA processing and modification
	GL25033	Glycosyl hydrolases family 18	Carbohydrate transport and metabolism
	GL17643	NA	Posttranslational modification, protein turnover, chaperones
	GL23829	Ser-Thr-rich glycosyl-phosphatidyl-inositol-anchored membrane family	Function unknown
	GL30186	Probable glycosidase	Carbohydrate transport and metabolism
	GL23083	NA	Function unknown

NA: not available.

Conversely, meiosis-related gene was not included in the “pink” module that exhibited a strong positive relationship with HZ2 (Fig. 5a and Supplementary Table 8). The hub genes in this module encoded the Ser-Thr-rich glycosyl-phosphatidyl-inositol-anchored membrane protein (GL23829), glycosyl hydrolase (GL25033), probable glycosidase (GL30186), caspase (GL15200), and 6 hypothetical proteins (GL21769, GL17422, GL26510, GL24251, GL17643, GL23083) (Table 3). DEGs in “pink” module were enriched into amino acid metabolism by KEGG, including the “tryptophan metabolism” and “arginine and proline metabolism” (Supplementary Fig. 5).

Using WGCNA, we found that the well-known meiosis-related genes associated with sporulation were presented in the “brown” module, which might be regulated by the TFs in the same module. Furthermore, the hub genes linked to energy production and signal transduction that might provide energy and signal molecules for meiosis and sporulation processes were identified by analyzing the coexpression networks.

## Discussion

*Ganoderma* spores are the germ cells ejected from *Ganoderma* gills and have high exploitable potential as the polysaccharide and oil extracted from the sporoderm-broken spores have been shown to be effective for the treatments of multiple diseases (Su et al. 2018; Jiao et al. 2020a, 2020b; Sang et al. 2021). However, few attentions have been paid to the basic biology of GLS. Since forward genetic dissection of basidiomycete mushrooms is a time-consuming task and sporulation-deficient mutants have never been reported in *G. lingzhi*, comprehensive analysis of factors and pathways that participate in spore morphogenesis using omics technologies has become an alternative option. Here, comparative transcriptomic analyses of 2 widely cultivated strains, HZ203 (low-sporing strain) and YW-1 (high-sporing strain), were performed to identify potential genes and metabolic pathways involved in basidiosporogenesis in *G. lingzhi*.

### Developmentally regulated transcriptional factors and their functions in sporulation

HZ203 produces normal but few spores compared with YW-1, which suggests that the sporulation process is normal in HZ203 and the limited spore number is not caused by a single recessive mutation, but by quantitative variations, including changes in the temporal or spatial expression pattern of relevant genes. It is well known that TFs act as key regulatory switches orchestrating spatiotemporally precise gene expression programs, essential for the proper control of growth and stress responses in all organisms (Lopes et al. 2021; Manna et al. 2021). In the present study, 7 transcriptional regulators were developmentally regulated in HZ203 and YW-1 and maintained the same expression tendency. As sporulation occurred normally in the 2 strains, we presumed that some of these differentially expressed TFs might be involved in the control of basidiosporogenesis in *G. lingzhi*. Meanwhile, GL23076, the C2H2-containing TF, is purportedly involved in cell cycle control, and its homolog SFP1 in *S.*  *pombe* regulates G2/M transitions during the mitotic cell cycle and DNA damage response (Xu and Norris 1998). Among the 14 strain-specific developmental-regulated TFs in HZ203, 12 were downregulated and their expression levels in YW-1 were similar to those at the HZ2 stage. The expression levels of 2 upregulated TFs were not significantly different between strains at the sporulating phase (Fig. 2b), which did not correspond to the phenotype. Consequently, we suggest that the transcriptional dynamics of these 14 TFs do not contribute to the phenotypic variation between YW-1 and HZ203, even though homologs of 3 TFs were reported to play a role in sporulation, including CON7 (GL21755) (Shi et al. 1998; Odenbach et al. 2007), BrlA (GL26016) (Adams et al. 1988), and CrzA (GL16023) (Dubey et al. 2016).

In contrast, 12 of 18 TFs in YW-1 were upregulated, and the expression patterns of *GL18912*, *GL24460*, *GL30967*, and *GL24474* were positive correlated with the spore number in each sample (Supplementary Table 6); in particular, GL24474 was homologous to the meiosis-specific protein HOP1 in *S.*  *cerevisiae* (Kariyazono et al. 2019). In addition, we performed gene coexpression network analysis to identify genes highly correlated with sporulation and calculate the correlation between genes. Results showed that *GL18912* had high connectivity with *MSH4* (*GL30856* and *GL30857*) and *Spo5* (G*L24035*). As MSH4 and Spo5 have been reported to promote the progression of meiosis and spore formation (Kasama et al. 2006; Okuda et al. 2013), and the expression levels of *GL30856*, *GL30857*, and G*L24035* were positively associated with spore number, we inferred that the C2H2-containing TF (GL18912) might interact with *MSH4* and *Spo5*, thereby regulating their expression to promote spore formation.

Recently, it was reported that carbohydrate catabolism is activated when spores are produced at a high level. For example, lignocellulolytic enzymes were upregulated, while glycogen, trehalose, and mannitol accumulated in the early phases decreased, suggesting that the sporulation process requires energy and intermediates produced from carbohydrate catabolism to synthesize the structural components (Zhou et al. 2021). Among the YW-1-specific regulated TFs, GL16151 and GL30300 might participate in carbohydrate metabolism. In *S.*  *cerevisiae*, when glucose becomes scarce, ethanol is used as a carbon source, which increases the expression levels of genes involved in gluconeogenesis and the glyoxylate cycle. ERT1 plays a role in activating the expression of genes involved in gluconeogenesis and mitochondrial function (Gasmi et al. 2014). *GL16151*, the homolog of *ERT1*, was upregulated in YW-1, and methane metabolism and the glyoxylate cycle were activated (Fig. 4a). These results indicated that GL16151 might also act as an activator of the glyoxylate cycle which produces NADH as an energy source. Without chloroplasts, *G. lingzhi* can only receive the energy and carbon sources from the substrate degradation products, which are produced by a series of efficient enzyme systems (Zhou et al. 2018). XlnR is a transcriptional activator of xylanolytic and cellulolytic genes in *Aspergillus*, which regulates the expression of hydrolytic genes for the degradation of β-1,4-xylan, arabinoxylan, cellulose, and xyloglucan (Marui et al. 2002; Noguchi et al. 2009). The expression level of the homolog of XlnR in *G. lingzhi*, *GL30300*, was increased nearly 10-fold in YW-1 but stably transcribed in HZ203 (Fig. 2b). The elevated expression level of *GL30300* might activate its target genes and hence contribute to substrate degradation.

### Hub genes in sporulation involved in signal transduction and energy production

In addition to the above TFs and meiosis-related genes, 10 hub genes in the “brown” module highly correlated with sporulation were identified and 5 genes were matched in at least 1 public database. These hub genes encode proteins that participate in energy production and carbohydrate metabolism, including substrate transporter (GL25109), glycosyl transferase (GL24498), and NADP-dependent alcohol dehydrogenase (GL25699). *G. lingzhi* produces some of the most efficient enzyme systems to degrade wood, and the degradation products, including mono- and oligosaccharides, are transported to the basidia and utilized in several carbohydrate metabolic pathways to support fruiting body development and secondary metabolism (Zhou et al. 2018). These 3 hub genes might participate in providing energy and structural material for spore formation and development, as reported recently (Zhou et al. 2021). Moreover, *GL29810* encodes a putative serine/threonine-protein kinase. Serine/threonine-protein kinases play a key role in signal transduction, including sensing the nutrient status of the cell and regulating cellular metabolism (DeMille and Grose 2013; Ben-Sahra and Manning 2017). For example, mTOR is an evolutionarily conserved serine/threonine-protein kinase that senses and integrates signals from intracellular nutrients (such as glucose and amino acids) and metabolic processes to stimulate cell growth (Jewell and Guan 2013; Ben-Sahra and Manning 2017). Although the biological function of *GL29810* in *G. lingzhi* remains unknown, it was highly correlated with genes involved in energy production and carbohydrate metabolism; therefore, we inferred that *GL29810* might also play a role in nutrient sensing and thereby regulate carbohydrate metabolism. Validation of the biological functions of these candidate genes requires further research.

### Difference in energy sources between high- and low-sporing genotypes

Coinciding with hub genes functions in energy production and carbohydrate metabolism, DEGs specific in YW-1 were enriched in “carbon metabolism” and “starch and sucrose metabolism” (Fig. 4); furthermore, genes in “brown” module were also enriched in glyoxylate and dicarboxylate metabolisms (Supplementary Fig. 5). Taken together, these results imply that the metabolic pathways involved in carbon substances and energy sources are pivotal for spore production. Similarly, increased carbohydrate supply was found in the highly spore-producing strain G0119 (Zhou et al. 2021).

Contrary to the carbohydrate metabolism in YW-1, developmentally regulated genes specific to HZ203 participated in amino acid metabolism and degradation, and all involved genes were downregulated (Fig. 4a). Amino acids are individual monomers that make up proteins, nucleotide bases, and other nitrogenous products, which are vital for maintaining glucose levels and providing alternative carbon sources during starvation (Judge and Dodd 2020). Proteases and amino acid metabolism were more active during sporulation in *Bacillus thuringiensis* (Wang et al. 2013), suggesting that spore formation is correlated with amino acid metabolism. In addition, the overlapping upregulated genes were enriched in the amino and nucleotide sugar metabolism, fatty acid metabolism, and unsaturated fatty acid biosynthesis by KEGG (Fig. 4a). Fatty acids not only act as membrane constituents but are also essential for energy provision. In summary, these results suggest that spore morphogenesis is correlated with energy metabolism; amino acid and fatty acid metabolisms might provide energy for this sexual process but could not meet the demand for high levels of spore production, which could be achieved by carbohydrate metabolism.

Triterpenoids are major bioactive compounds in *G. lingzhi*, which are key determinants of fruiting body quality. However, it has been reported that triterpenoid content is clearly reduced from primordia to mature fruiting bodies (Chen et al. 2012). Triterpenoid synthesis is suggested to be triggered by carbohydrate metabolism as acetyl-CoA, the precursor for the synthesis of triterpenoids, is an intermediate of carbohydrate metabolism (Zhou et al. 2021). Consistent with this suggestion, we found that the expression levels of several key enzyme genes involved in the biosynthesis of triterpenoids were downregulated at the sporulating phase, compared to the young fruiting body without spores. Furthermore, these genes were transcribed at a higher level in HZ203 than that in YW-1. High expression of these important biosynthetic genes has been shown to increase ganoderic acid and the by-product (ergosterol) content (Shi et al. 2012; Xu et al. 2010, 2012; Fei et al. 2019), suggesting that these metabolites might accumulate at higher levels in HZ203. Taken together, we suggest that spore morphogenesis and development take advantage of the energy and intermediate from carbohydrate metabolism, which reduces the carbon source from triterpenoid biosynthesis. Therefore, developing a low-sporing or sporulation-deficient strain might be useful for improving the quality of fruiting bodies, which requires further research.

## Conclusion

In this study, we performed comparative transcriptomics at 2 developmental stages (before and after sporulation) of 2 strains to explore the genetic regulatory mechanism of sporulation in *G. lingzhi*. Combined GO term, KEGG, and WGCNA analysis identified candidate genes and purported pathways associated with sporulation. We speculated that *GL30856* and *GL30857* (homolog of MSH4), *GL24035* (homolog of Spo5), *GL24474* (homolog of HOP1), and *GL18912* (C2H2-containing TF) are candidate genes for spore formation, and *GL18912* might regulate the expression levels of *GL30856*, *GL30857*, and GL*24035*; *GL16151* (Zn2Cys6-containing TF) and *GL30300* (Zn2Cys6-containing TF) were potential genes for activating carbohydrate metabolism. Furthermore, the energy and structural components required for sporulation may be supported by the carbohydrate metabolism pathway.

## Conflicts of interest

The authors declare that the research was conducted in the absence of any commercial or financial relationships that could be construed as a potential conflict of interest. The authors declare no conflict of interest.

## Data availability

The data presented in this study were deposited into the Sequence Read Archive database (accession numbers: PRJNA704770 and PRJNA736534).

Supplemental material is available at *G3* online.

## Supplementary Material

jkab448_Supplementary_Figure_S1

jkab448_Supplementary_Figure_S2

jkab448_Supplementary_Figure_S3

jkab448_Supplementary_Figure_S4

jkab448_Supplementary_Figure_S5

jkab448_Supplementary_Table

jkab448_Supplemental_Material_Legends
