# Supplementary figures and images for: Comparative transcriptome analysis of genes and metabolic pathways involved in sporulation in Ganoderma lingzhi
Source: G3 (Bethesda). 2022 Jan 17;12(3):jkab448. doi: 10.1093/g3journal/jkab448 (PMC8895980; doi:10.1093/g3journal/jkab448)

A

HZ203

1mm

B

YW-1

1mm

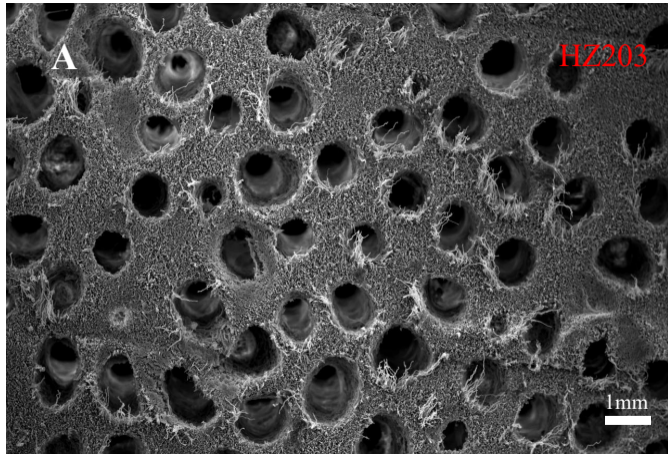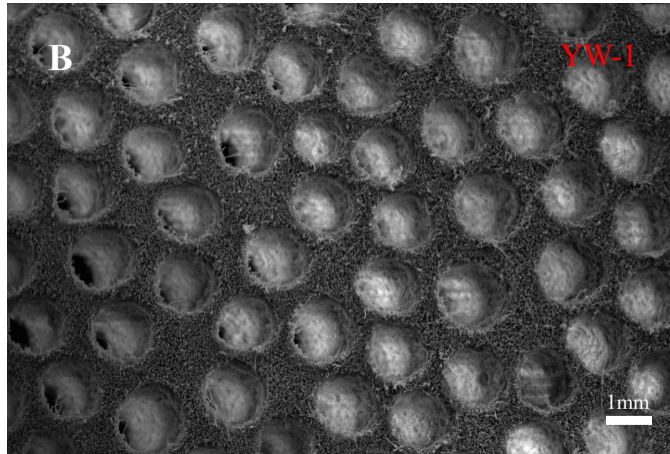

Supplement: jkab448_Supplementary_Figure_S1 [file jkab448_supplementary_figure_s1.pdf]

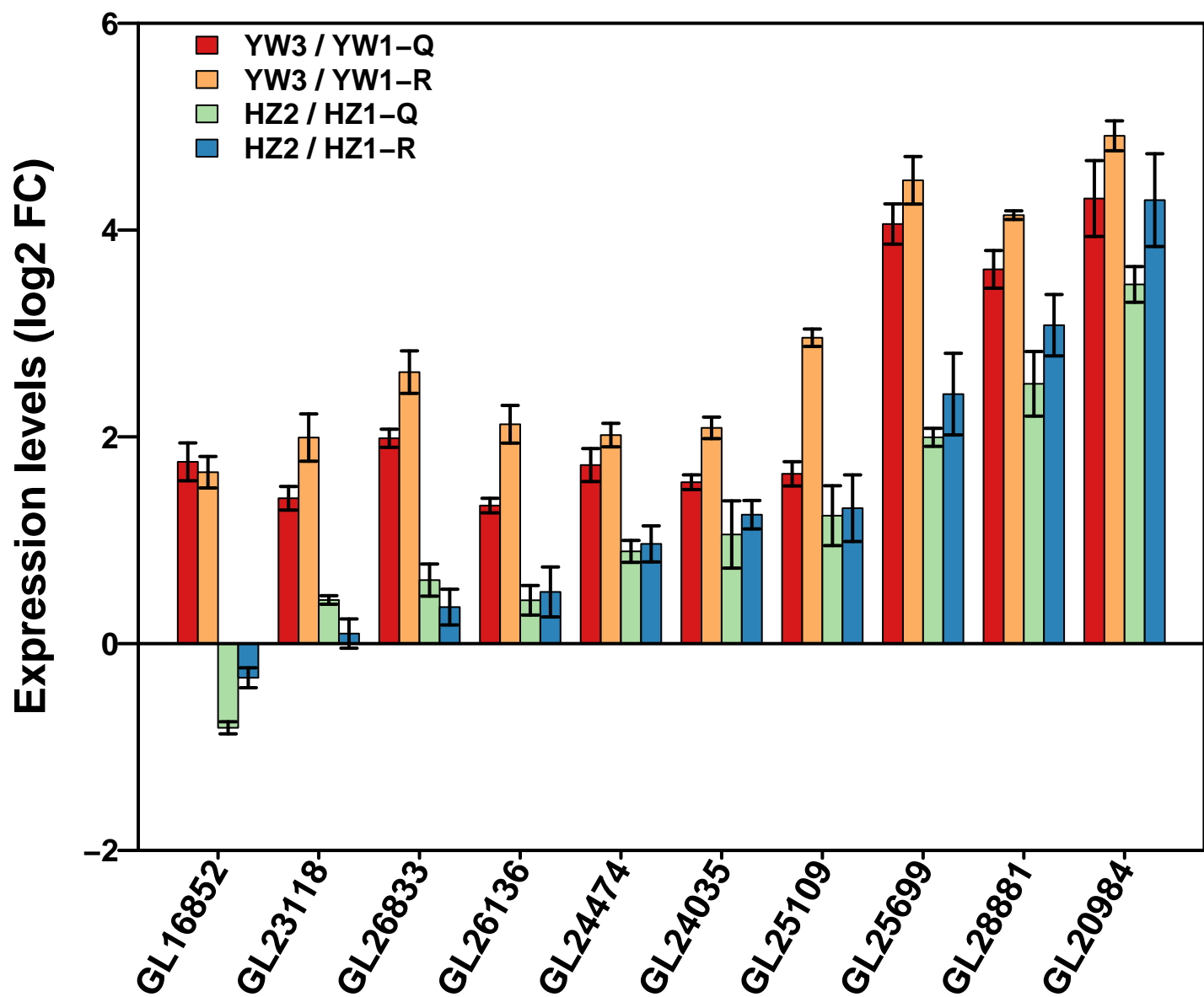

Supplement: jkab448_Supplementary_Figure_S2 [file jkab448_supplementary_figure_s2.pdf]

**A**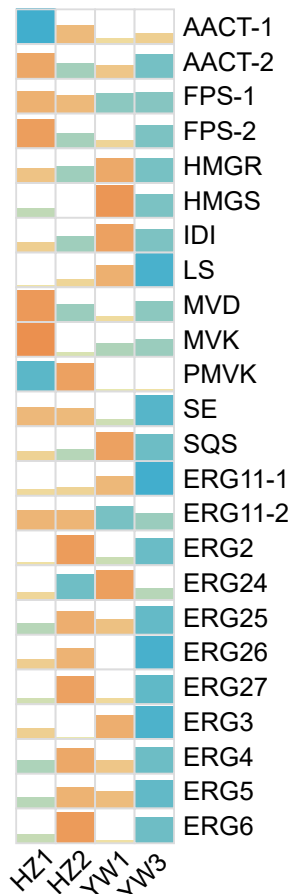**B**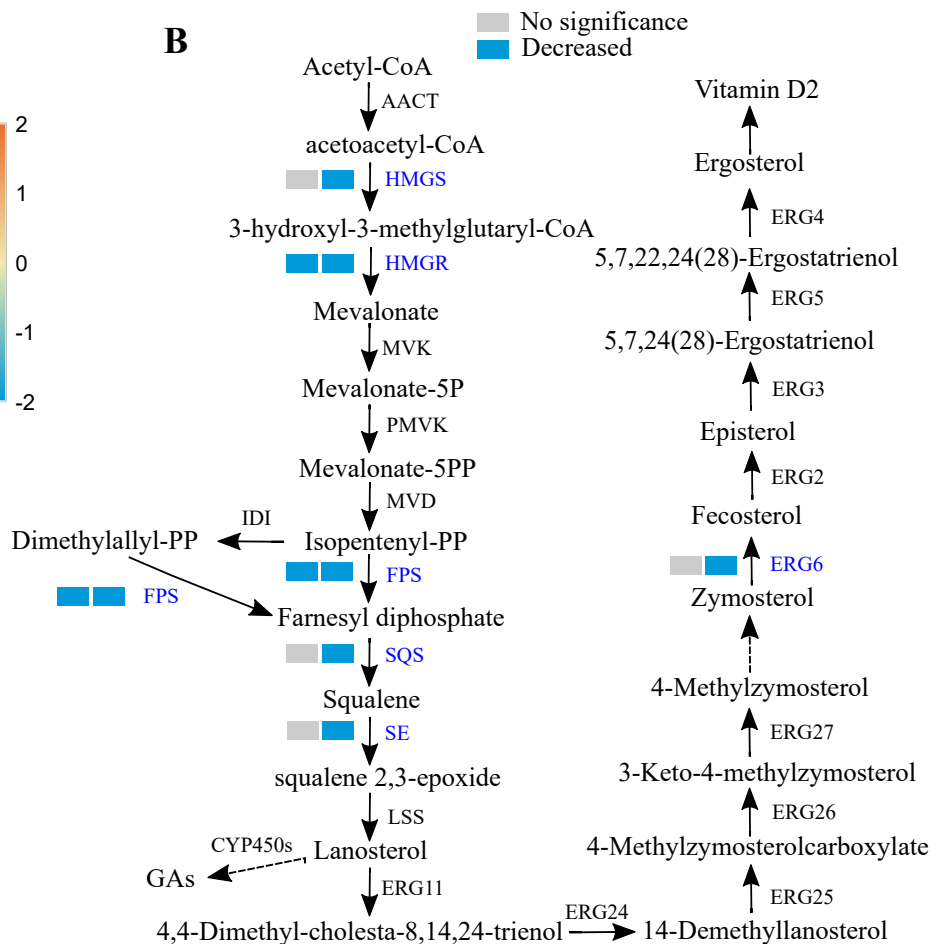

Supplement: jkab448_Supplementary_Figure_S3 [file jkab448_supplementary_figure_s3.pdf]

Cluster Dendrogram

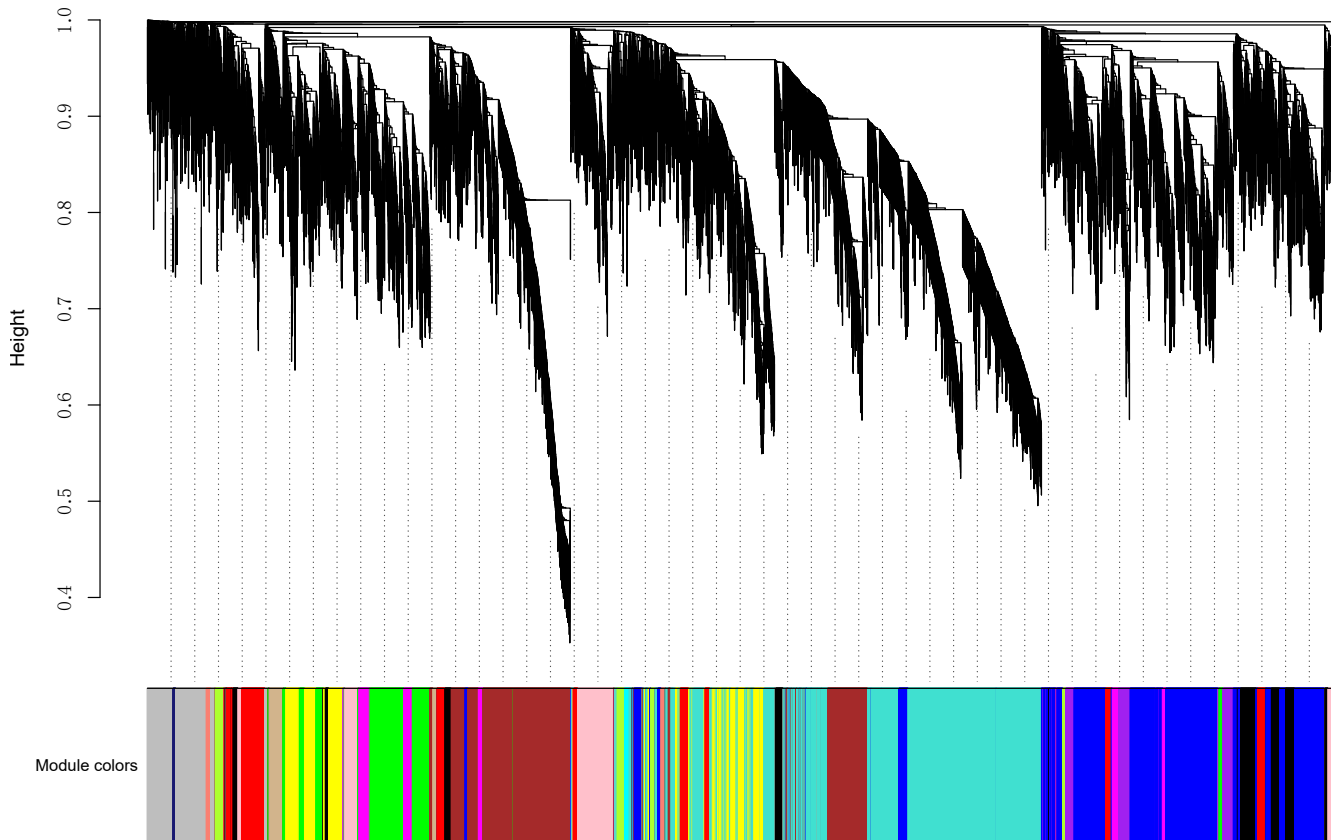

Supplement: jkab448_Supplementary_Figure_S4 [file jkab448_supplementary_figure_s4.pdf]

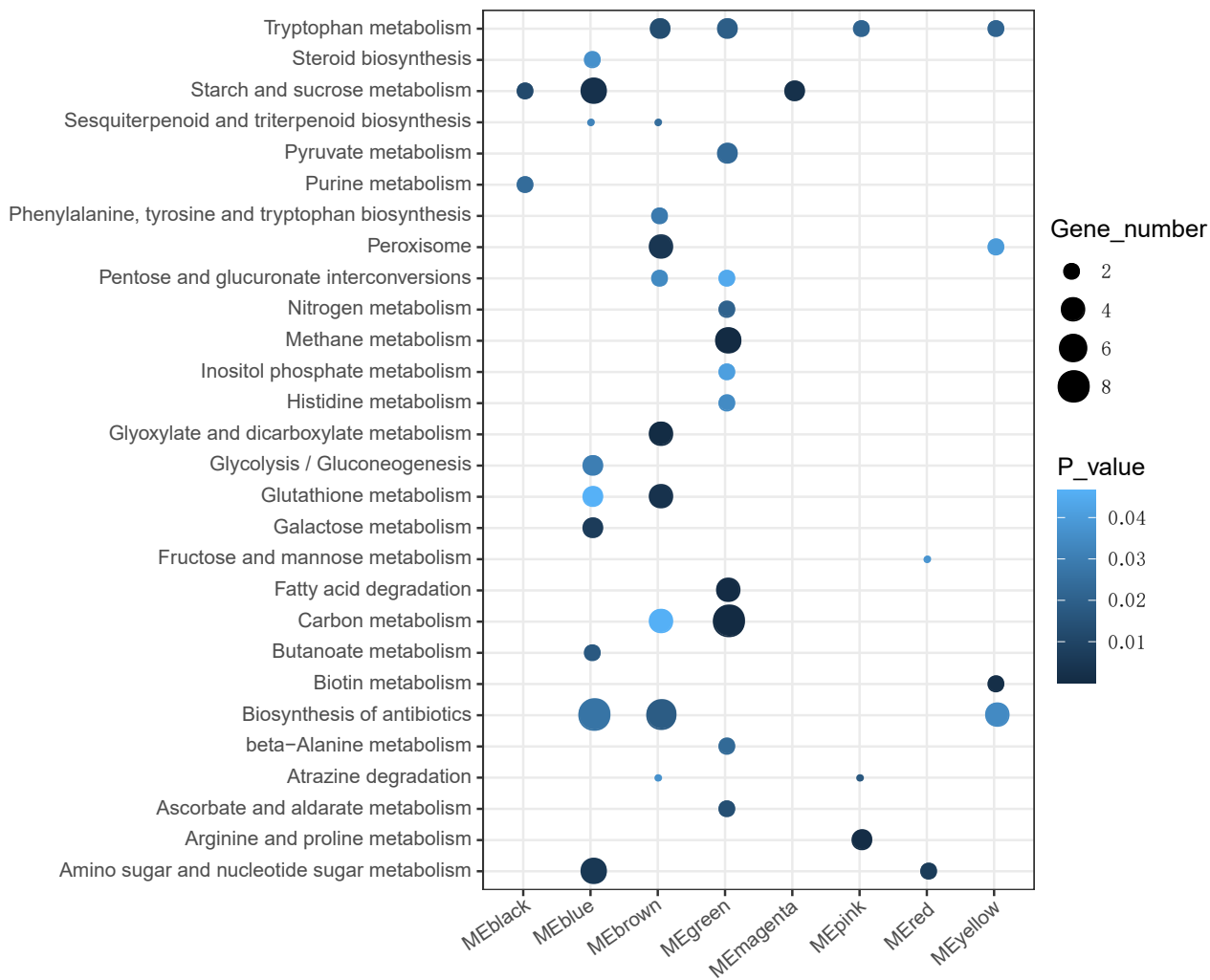

Supplement: jkab448_Supplementary_Figure_S5 [file jkab448_supplementary_figure_s5.pdf]
