# Supplementary material for: Comparative transcriptome analysis of genes and metabolic pathways involved in sporulation in Ganoderma lingzhi
Source: G3 (Bethesda). 2022 Jan 17;12(3):jkab448. doi: 10.1093/g3journal/jkab448 (PMC8895980; doi:10.1093/g3journal/jkab448)
Supplement: jkab448_Supplemental_Material_Legends [file jkab448_supplemental_material_legends.docx]

**Supporting information**

Supplementary Figure 1. Pore morphology in YW-1 and HZ203.

Supplementary Figure 2. Expression level of selected genes in the low sporing strain HZ203 and the high sporing strain YW-1 analysed by qRT-PCR. qRT-PCR verification of the expression of ten differentially expressed genes (DEGs) in two stages. Q: qRT-PCR; R: RNA-seq.

Supplementary Figure 3. Genes predictably involved in triterpenoid and ergosterol biosynthesis pathways. (A) Heatmap shows the expression level of the genes coding for key enzymes, which are predictably involved in triterpenoid and ergosterol biosynthesis pathways. (B) A schematic of triterpenoid and ergosterol biosynthesis pathways in *Ganoderma lingzhi*.

Supplementary Figure 4. Genes cluster dendrogram (hierarchical clustering tree) of the transcriptome.

Supplementary Figure 5. Kyoto Encyclopedia of Genes and Genomes (KEGG) pathway enrichment of differentially expressed genes in different modules. Significantly enriched pathways with *P* < 0.05 are shown.

Table S1 Primers used for qRT-PCR analysis of selected genes.

Table S2 Summary of Illumina transcriptome sequencing for *Ganoderma lingzhi*.

Table S3 List of new genes identified in HZ203 strain as compared to G.260125-1.

Table S4 Numbers of detected transcripts in each sample.

Table S5 List of differentially expressed genes between developmental stages of the *Ganoderma lingzhi* basidiospore.

Table S6 Summary of the differential expressed transcription regulators.

Table S7 Summary of the differential expressed genes in carbon metabolism pathway.

Table S8 Summary of the differential expressed genes in each module.

Table S9 Summary of the connectivity of differentially expresses genes in each module.
